# Overexpression of human sperm protein 17 increases migration and decreases the chemosensitivity of human epithelial ovarian cancer cells

**DOI:** 10.1186/1471-2407-9-323

**Published:** 2009-09-11

**Authors:** Fang-qiu Li, Yan-ling Han, Qun Liu, Bo Wu, Wen-bin Huang, Su-yun Zeng

**Affiliations:** 1 Laboratory of Molecular Biology, Institute of Medical Laboratory Sciences, Jinling Hospital, School of Medicine, Nanjing University, Nanjing 210002, PR China; 2 Department of Pathology, Jinling Hospital, School of Medicine, Nanjing University, Nanjing 210002, PR China; 3 Department of Gynecology, Jinling Hospital, School of Medicine, Nanjing University, Nanjing 210002, PR China

## Abstract

**Background:**

Most deaths from ovarian cancer are due to metastases that are resistant to conventional therapies. But the factors that regulate the metastatic process and chemoresistance of ovarian cancer are poorly understood. In the current study, we investigated the aberrant expression of human sperm protein 17 (HSp17) in human epithelial ovarian cancer cells and tried to analyze its influences on the cell behaviors like migration and chemoresistance.

**Methods:**

Immunohistochemistry and immunocytochemistry were used to identify HSp17 in paraffin embedded ovarian malignant tumor specimens and peritoneal metastatic malignant cells. Then we examined the effect of HSp17 overexpression on the proliferation, migration, and chemoresistance of ovarian cancer cells to carboplatin and cisplatin in a human ovarian carcinoma cell line, HO8910.

**Results:**

We found that HSp17 was aberrantly expressed in 43% (30/70) of the patients with primary epithelial ovarian carcinomas, and in all of the metastatic cancer cells of ascites from 8 patients. The Sp17 expression was also detected in the metastatic lesions the same as in ovarian lesions. None of the 7 non-epithelial tumors primarily developed in the ovaries was immunopositive for HSp17. Overexpression of HSp17 increased the migration but decreased the chemosensitivity of ovarian carcinoma cells to carboplatin and cisplatin.

**Conclusion:**

HSp17 is aberrantly expressed in a significant proportion of epithelial ovarian carcinomas. Our results strongly suggest that HSp17 plays a role in metastatic disease and resistance of epithelial ovarian carcinoma to chemotherapy.

## Background

Ovarian cancer is the fifth or sixth most common cause of gynecologic cancer morbidity and mortality in developed countries [1]. Most deaths from ovarian cancer are caused by metastases that are resistant to conventional therapies. Although ovarian cancers metastasize primarily by exfoliation followed by peritoneal implantation, about 40% of patients with advanced ovarian cancer show lymph node metastasis and/or extra-abdominal metastasis. The factors that regulate the metastatic process and chemoresistance of ovarian cancer remain poorly understood.

Recent studies suggest that the enhanced expression and activation of matrix metalloproteinases MMP-2 and MMP-9 may play a role in ovarian cancer cell migration [2]. But other factors may also contribute to this malignant behavior. One candidate is human sperm protein 17 (HSp17), a normal tissue specific protein detected in the primary tumor cells from 70% of the patients with ovarian carcinoma [3].

HSp17 is a protein originally reported to be expressed exclusively in the testis and whose primary function is binding to the zona pellucida [4]. In addition to testis and ejaculated sperm, HSp17 was also detected in ciliated epithelia of the respiratory airways and both the male and female reproductive systems [5]. Recent evidence suggested that HSp17 was aberrantly expressed in many malignant tumors, such as multiple myelomas [6], ovarian cancers [7], esophageal squamous cell carcinomas [8], esthesioneuroblastomas [9], and various histological subtypes of human nervous system (NS) malignancies, including neuroectodermal and meningeal tumors [10]. Wang *et al*. showed that promoter methylation can serve as the main regulatory mechanism for the expression of HSp17 in tumor cell lines [11]. Because the function of the HSp17 in cancer cells is not known, we presume that this protein may play a role in the function of these ciliated cells and may influence the malignant behaviors of ovarian cancer cells.

In this study, we investigated the frequency of HSp17 expression at the protein level and its cell pattern distribution in various histological subtypes of human ovarian tumors by immunohistochemistry. We then examined the effect of HSp17 overexpression on the migration of an ovarian cancer cell line, HO8910, and its chemoresistance to cisplatin and carboplatin by introducing HSp17 cDNA into the cells.

## Methods

### Patient specimens

A panel of formalin-fixed paraffin-embedded ovarian tumor specimens were obtained from the archival resource of Department of Pathology, Jinling Hospital, consisting of 70 epithelial ovarian carcinomas (EOC), (median) age 52 (22~80), and 7 non-epithelial malignant tumors primarily developed in the ovaries (1 germ cell tumor, 2 malignant mesodermal mixed tumors, 4 sex cord-stromal tumors), (median) age 38 (25~55). All tumors were classified according to WHO criteria. HSp17 expression in metastatic cancer cells was measured in ascitic fluids obtained from 8 of the EOC patients, and in metastatic tumor tissues obtained from 10 patients with metastatic ovarian cancer in endometrium, oviduct, omentum majus, rectal sinuses, vagina and jejunoileum. Specimen collection and archiving of patient data was performed with written informed consent and approved by the ethical committee of the hospital.

### Immunohistochemistry

Tissue sections (3 μm) were placed on glass slides, heated at 60°C for 20 min, and then deparaffinized with xylene and ethanol. For antigen retrieval, tumor specimens mounted on glass slides were immersed in preheated antigen retrieval solution (DAKO high pH solution, DAKO) for 20 min and allowed to cool for 20 min at room temperature. After the inactivation of endogenous peroxidase, anti-HSp17 monoclonal antibody (purchased from BD Bioscience Co, clone 21) was added at a concentration of 2 μg·ml^-1 ^and incubated overnight at 4°C. The primary antibody was detected with an anti-mouse IgG (DAKO). Diaminobenzidine (DAB) substrate was added for 7 min followed by washing with deionized water, and hematoxylin was applied for 1 min to counterstain the tissue sections. The tissue sections were dehydrated via graded ethanols followed by xylene, and coverslips were attached with permount. The extent of immunohistochemical reactivity was graded as follows: negative; +, 5-25% of malignant cells stained; + +, >25-50% of malignant cells stained; + + +, >50-75% of malignant cells stained; and + + + +, >75% of malignant cells stained. Two independent reviewers scored it and results were in consensus. Negative control slides omitting the primary antibody were included in all assays.

### Immunocytochemistry

To investigate the expression of HSp17 in ascetic metastatic tumor cells, washed cells were cytocentrifuged for 3 min at 800 rpm on a glass slide using a Cytospin-2 centrifuge and then fixed with Biofix (Bio-Optica; Milan, Italy), then treated with anti-Sp17 antibody with the same protocol as immunohistochemistry.

### Cell line and culture conditions

The HO8910 human ovary carcinoma cell line was a gift from Dr. Liu Qi (Medical School of Nanjing University, China). It was cultured in DMEM supplemented with 10% fetal bovine serum (FBS) and penicillin-streptomycin at 37°C in a 5% CO_2 _incubator. *In vitro *experiments were done at 70% to 80% confluence. Results from all studies were confirmed in at least three independent experiments.

### Establishment of cell model with overexpression of HSp17

For experiments regarding the roles of HSp17 in cancer migration and chemoresistance, an HSp17 and GFP co-expression vector was constructed and introduced into the EOC cell line HO8910 in which HSp17 is not expressed. HSp17 cDNA was amplified by polymerase chain reaction (PCR) from a plasmid pGEM-T/hSp17 which was constructed in our laboratory using human testicular cDNA and used for generating recombinant HSp17 protein [12]. Purified HSp17 cDNA and green fluorescent protein-producing pEGFP-N1 vector were digested by *HindIII*/*KpnI*, and then ligated together by T4 ligase to generate the recombinant plasmid pEGFP-N1/hSp17. 5 × 10^5 ^HO8910 cells were seeded in each well of a 12-well plate and transfection was conducted when the cells grew to 70% confluence. In vitro DNA transfection was by lipofectamine-mediated transfection procedure. The cells were incubated at 37°C under an atmosphere of 5% CO_2 _and 6 h later, the media was replaced with DMEM containing 10% FCS. Stably transfected clones were selected 36 h later by adding G418 (Roche Molecular Biochemicals) at 500 μg·ml^-1^. The stable clones were then evaluated for the expression of hSp17/EGFP by fluorescent microscopy, and confirmed by Western blot and immunohistochemistry analysis. Transfectants with vector pEGFP-N1/hSp17 and pEGFP-N1 were termed HO8910/hSp17 and HO8910/EGFP cells, respectively.

### Western blotting

Proteins in HSp17/EGFP expressing cells and EGFP expressing cell lysates (20 μg protein/lane) were separated by sodium dodecyl sulfate-polyacrylamide gel electrophoresis (SDS-PAGE) in 12% gels and then transferred to a nitrocellulose membrane (Amersham Biosciences, Piscataway, NJ). After the blot was blocked in PBS-T with 5% skim milk for 1 h at 37°C, membrane-bound proteins were immunolabeled with anti-HSp17 monoclonal antibody overnight at 4°C. The membranes were washed three times. The horseradish peroxidase-conjugated rabbit anti-mouse IgG antibody was incubated with the membranes for 1 h at RT and subsequently washed three times. Membranes were then detected with the enhanced chemiluminescence method (Santa Cruz Biotechnology, CA) and x-ray film. Purified recombinant HSp17 protein generated in our laboratory [12] was used as a positive control.

### In vitro migration assay

Cell migration was examined using Transwell chamber assay according to the protocol of the manufacturer (Becton Dickinson Labware, Bedford, MA). Briefly, 2 × 10^5 ^HO8910/hSp17 and HO8910/EGFP cells in DMEM plus 1% FBS were placed on each 8.0-μm pore size membrane insert in 24-well plates. DMEM plus 10% FBS was placed in the bottom wells as chemoattractants. After 20 h, cells that did not migrate were removed from the top side of the inserts with a cotton swab. Cells that had migrated to the underside of the inserts were stained with crystal violet and the cells on each insert were counted at 200× magnification.

### MTT cell viability assay

The HO8910/hSp17 and HO8910/EGFP cells were seeded in 96-well plates (Costar; Corning Inc., Corning, NY) for 24 h and then exposed to various concentrations of cisplatin or carboplatin (QiLu Pharma. Co. China) for 48 h. The cells were incubated with 10 μl of 2 mg·ml^-1 ^3-(4,5-dimethyl-2-thiazolyl)-2,5-diphenyl-2H-tetrazolium bromide (MTT, purchased from Sigma Co) in phosphate-buffered saline (PBS) for 4 h, and the formazan crystals were lysed with 150 μl of DMSO (dimethyl sulfoxide, from Sigma Co) for 10 min at 37°C. Absorbance at 570 nm was then measured with a microplate reader (Bio-Rad, 680, Hercules, CA). Normal cell culture and no cell wells were set as control. All concentrations were tested in triplicates. Absorbance values were translated to inhibition rate by the equation 1-(OD of test concentration-OD of no cell wells)/(OD of control-OD of no cell wells).

### Statistical analysis

All statistical analyses were performed with the SPSS 13.0 software. Differences in proportions were evaluated by the Fisher's exact test as appropriate. One-way ANOVA was used for mean comparison. Results were considered statistically significant at the *P *< 0.05 level.

## Results

### Immunohistostaining of HSp17

**Figure 1:**
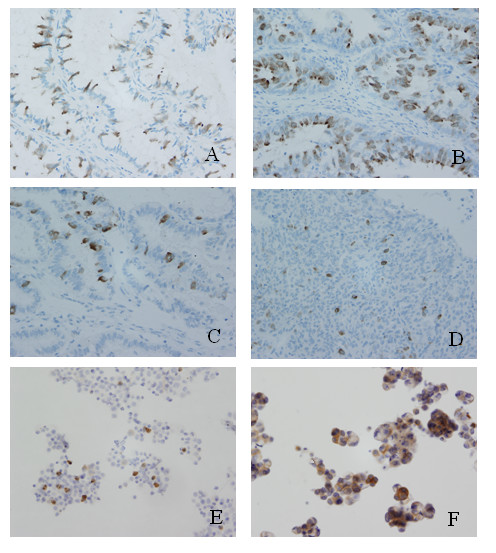
**Immunohistochemistry and immunocytochemistry staining of HSp17 in epithelial ovarian carcinomas**. A. diffuse expression in mucus palillary cystadenocarcinoma; B. diffuse expression in serous palillary adenocarcinoma; C. partis expression of HSp17 in serous palillary adenocarcinoma; D. scattered expression in poorly differentiated ovarian serous palillary cystadenocarcinoma; E, F. cells from ascitic fluids. (original magnification, 200×).

A total of 77 archival ovarian malignancy specimens were investigated by immunohistochemistry. Positive staining was observed in 43% (30/70) of epithelial ovarian cancers. HSp17 protein was expressed in the adenocarcinoma cells of all subtypes of epithelial ovarian cancer (Table 1). The expression patterns of HSp17 we observed in EOC were always heterogeneous (Figure 1). In contrast to previous reports [7,8], we found HSp17 mainly localized in the cytoplasm of a variable number of tumor cells, but positive staining of the cell surface was also detected. No reactivity was noted in non-epithelial malignant tumors. All of the metastatic ascitic fluid cancer cells from the 8 EOC patients were positive for HSp17 (Figure 1). The Sp17 expression was also detected in the metastatic lesions was the same as in ovarian lesions.

**Table 1:** HSp17 expression in EOC by IHC

	Negative	Positive	+	+ +	+ + +	+ + + +
All tumors, n (%) (n = 70)	40	30(43%)	10(14.3%)	6(8.6%)	9(12.9%)	5(7.1%)
Serous	22	15	5	3	3	4
Mucinous	11	6	3	2	1	0
Primary peritoneal carcinoma	3	4	1	1	2	0
Clear cell	2	2	1	0	1	0
Endometroid	2	2	0	0	2	0
Undifferentiated	0	1	0	0	0	1

-, 5%; +, <25%; ++, 25-50%; +++, 50-75%; ++++ >75%.

### Co-expression of Sp 17 and GFP in HO8910 cells

**Figure 2:**
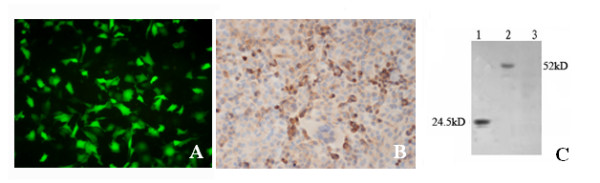
**Co-expression of Sp17 with EGFP in HO8910/hSp17 cells**. A. fluorescence microscopy showing expression of GFP. B. immunocytochemistry showing expression of Sp17 in HO8910 cells (original magnification, 200×). C. Western blot analysis: 1. recombinant HSp17; 2. lysate of HO8910/hSp17 cells; 3. lysate of HO8910/EGFP cells.

The co-expression of Sp17 and GFP in HO8910/hSp17 cells was observed under a fluorescence microscope (Figure 2A) and confirmed by immunocytochemistry with anti-HSp17 antibody (Figure 2B). In dividing cells, HSp17 is mostly localized to the cytoplasm and then shifts to the cell surface (Figure 2B). Western blotting analysis showed that the fusion protein of Sp17 and GFP was 52 kD, which is much larger than recombinant HSp17 (Figure 2C, 52 kD and 24.5 kD, respectively).

### Sp17 increased the migratory capacity of cells

**Figure 3:**
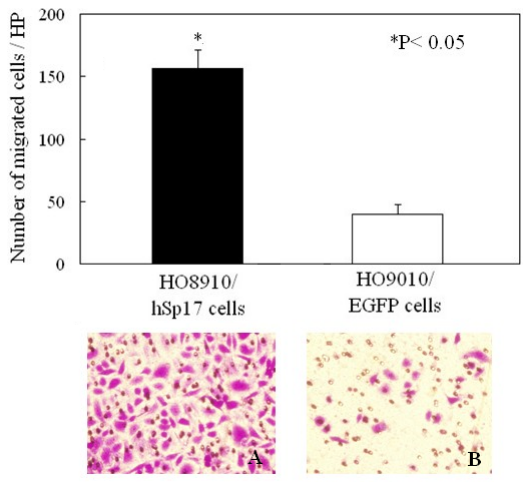
**HO8910/hSp17 cells have increased migratory capacity**. Transwell chamber assay was done to compare the migratory capabilities of HO8910/hSp17 (A) and HO8910/EGFP (B) cells. At 20 h, HO8910/hSp17 cells showed a ~3.2-fold increase in the number of cells migrating through the membrane (original magnification, 200×).

Transwell chamber assays were performed to compare the migratory capability of HO8910/hSp17 and HO8910/EGFP cells. At 20 h, the HO8910/Sp17 cells showed a ~3.2-fold increase in the number of cells migrating through the polycarbonate membrane (156.6 ± 14.9/HP for HO8910/hSp17 cells, 39.3 ± 8.53)/HP for HO8910/EGFP cells, n = 10, P < 0.05; Figure 3).

### Effect of Sp17 on chemoresistance

**Figure 4:**
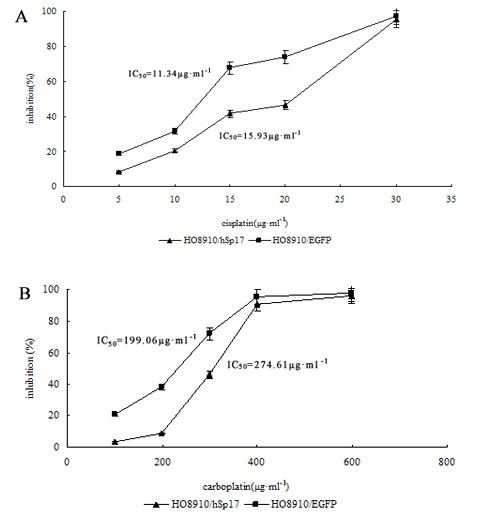
**HO8910/hSp17 and HO8910/EGFP cells are inhibited by cisplatin and carboplatin differently**. HO8910/hSp17 had higher viability than the control cells under different concentrations of cisplatin (A) and carboplatin (B), and the difference is quite significant (*p < 0.05, ** p < 0.05). Bars, SD.

The effects of cisplatin and carboplatin on the growth of HO8910/hSp17 cells and HO8910/EGFP cells were evaluated by measuring cell viability under different concentration of drugs using the MTT assay. The results indicated that HSp17 expression did not affect the viability of HO8910 cells that were not treated with drugs. On the other hand, the resistance to cisplatin and carboplatin was increased in HSp17 transfectants. The cells transfected with pEGFP-N1 were much more sensitive to the drugs (Figure 4). This effect was more obvious when the drug concentrations were between 15 μg·ml^-1^and 20 μg·ml^-1 ^for cisplatin, 100 μg·ml^-1 ^and 300 μg·ml^-1 ^for carboplatin. These results strongly suggest that Sp17 participates in the chemoresistance of ovarian carcinoma without influencing cell proliferation.

## Discussion

Previous studies have demonstrated the aberrant expression of some normal testicular proteins in neoplastic cells. These proteins collectively form a new class of tumor associated antigens called cancer-testis (CT) antigens [13,14]. Furthermore they have become the most extensively studied antigen group in the field of tumor immunology [12,15-17]. HSp17 is a highly conserved mammalian protein that was originally isolated from rabbit epididymal sperm and testis membranes and has been identified as a member of CT antigen family. The mRNA encoding HSp17 has been found to be highly expressed in a number of in vitro neoplastic cell lines, including lung cancer, prostate cancer, and osteocarcinoma [18]. HSp17 protein has been recognized in healthy testis [19], normal ciliated cells of the male and female reproductive tract [5], human multiple myeloma cell lines [20], and in malignant lymphocytes and various tumoral tissues [6-10]. The immunogenicity and tissue localization of Sp17 made it an attractive candidate for developing specific active immunotherapy procedures. Nakazato et al. identified Sp17 as a candidate gene related to the chemoresistance of clear cell carcinoma [21]. Bumm et al. showed that Sp17 can be used as a marker to discriminate between 2 subsets of primary esthesioneuroblastomas [9].

Our program's primary focus is to study the clinical relevance of aberrant Sp17 expression in order to identify a better prognostic marker and new treatment for ovarian cancer. We analyzed the expression of Sp17 in a panel of ovarian tumor tissues by immunohistochemistry and showed that Sp17 was positive in 43% percent of EOC specimens. We found that Sp17 was present in various tumor types of EOC. Its expression pattern was heterogeneous in all of the positive cancer tissue samples, and expression did not correlate with the histological subtype and degree of malignancy. Although late stage (III-IV) tumors expressed higher amount of Sp17 than early stage tumors, the statistical analysis did not show a significant difference (data not shown).

Unexpectedly, we observed that all of metastatic ascitic fluid cancer cells from eight EOC patients were positive for Sp17. Therefore, we presume that Sp17 could promote the migration and metastatic activity of cancer cells. In order to confirm this hypothesis, we generated a cell model that over-expressed Sp17 by transfecting Sp17 cDNA into an ovarian cancer cell line, HO8910. HSp17 was present in the cytoplasm and on the cell surface in these model cells, and induced a significant increase in HO8910 migration by a transwell assay. Previous reports [22] and our results show that the HSp17 gene is greatly induced in metastatic cells. Several recent reports have implicated Sp17 as having a role in cell-cell adhesion and/or cell migration in transformed, lymphocytic, and hematopoietic cells, possibly through its interaction with extracellular heparan sulphate [23,24] or its binding to AKAP3.

Although investigators in different laboratories observed Sp17 in the nucleus, cytoplasm, and on the cell surface, little knowledge of its function related to its localization has been reported. The role of Sp17 in promoting heparin sulphate-mediated adhesion of lymphoid cells has been proposed by Lacy and Sanderson [23], who showed that Sp17 expressed on the surface of lymphoid-derived cells from a patient with plasma cell leukemia promoted cell-cell adhesion via interaction with the heparin sulphate chain of syndecan 1.

Among the three functional domains of HSp17 [25,26], the N-terminal domain is similar to the dimer-interaction site in the cAMP-dependent protein kinase A (PKA) IIa regulatory subunit (RII alpha), the central sulphated carbohydrate-binding domain is necessary for heparin binding. Sp17's binding to A-kinase anchoring protein 3 (AKAP3) may reflect a functional need for Ca^2+^/CaM at sites along the flagella, where Ca^2+^/CaM is known to play a role in motility.

Nakazato et al. showed that Sp17 is differentially expressed between clear cell carcinoma and serous adenocarcinoma of the ovary. They examined the effect of small interfering RNA targeting the Sp17 gene on the chemoresistance of clear cell adenocarcinoma of the ovary to paclitaxel and found that this treatment decreased the chemoresistance of these cells to paclitaxel [21]. Their study suggests that expression of the Sp17 gene can be used as a predictor of the chemoresistance of ovarian cancer to paclitaxel. We also demonstrated that Sp17 plays an important role in the chemoresistance of the ovarian cancer cell line HO8910 to cisplatin and carboplatin, the other two fist-line chemotherapeutics for ovarian cancer. Although further investigation is needed to clarify the functions of Sp17 in ovarian cancer, our study suggests that expression of the Sp17 gene can be used as a predictor of the migration and chemoresistance of ovarian cancer to chemotherapeutics.

In conclusion, we investigated the aberrant expression of human sperm protein 17 in human epithelial ovarian cancer cells and its influences on the cell migration and chemoresistance, and found that HSp17 was aberrantly expressed in 43% of the patients with primary epithelial ovarian carcinomas, and in all of the metastatic cancer cells of ascites from 8 patients; Overexpression of HSp17 increased the migration but decreased the chemosensitivity of ovarian carcinoma cells to carboplatin and cisplatin. The results strongly suggest that HSp17 plays a role in metastatic disease and resistance of epithelial ovarian carcinoma to chemotherapy.

## Competing interests

The authors declare that they have no competing interests.

## Authors' contributions

FQL conceived, coordinated and designed the study, and contributed to the acquisition, analysis and interpretation of data and drafted the manuscript. YLH and QL performed the experiment and involved in drafting the article. BW and WBH performed selection of archived samples and scored the immunohistochemistry staining. SYZ participated in sample collection and data acquisition. All of the authors have read and approved the final manuscript.

## Pre-publication history

The pre-publication history for this paper can be accessed here:

http://www.biomedcentral.com/1471-2407/9/323/prepub
